# Sodium and potassium excretion in an adult Caribbean population of African descent with a high burden of cardiovascular disease

**DOI:** 10.1186/s12889-018-5694-0

**Published:** 2018-08-09

**Authors:** Rachel M. Harris, Angela M. C. Rose, Ian R. Hambleton, Christina Howitt, Nita G. Forouhi, Anselm J. M. Hennis, T. Alafia Samuels, Nigel Unwin

**Affiliations:** 1 The George Alleyne Chronic Disease Research Centre, Caribbean Institute for Health Research, The University of the West Indies, Bridgetown, Barbados; 2 MRC Epidemiology Unit, University of Cambridge, Cambridge, UK

**Keywords:** 24-h urine collection, Adult, Barbados, Diet, Excretion, Potassium, Salt, Sodium

## Abstract

**Background:**

High sodium diets with inadequate potassium and high sodium-to-potassium ratios are a known determinant of hypertension and cardiovascular disease (CVD). The Caribbean island of Barbados has a high prevalence of hypertension and mortality from CVD. Our objectives were to estimate sodium and potassium excretion, to compare estimated levels with recommended intakes and to identify the main food sources of sodium in Barbadian adults.

**Methods:**

A sub-sample (*n* = 364; 25–64 years) was randomly selected from the representative population-based Health of the Nation cross-sectional study (*n* = 1234), in 2012–13. A single 24-h urine sample was collected from each participant, following a strictly applied protocol designed to reject incomplete samples, for the measurement of sodium and potassium excretion (in mg), which were used as proxy estimates of dietary intake. In addition, sensitivity analyses based on estimated completeness of urine collection from urine creatinine values were undertaken. Multiple linear regression was used to examine differences in sodium and potassium excretion, and the sodium-to-potassium ratio, by age, sex and educational level. Two 24-h recalls were used to identify the main dietary sources of sodium. All analyses were weighted for the survey design.

**Results:**

Mean sodium excretion was 2656 (2488–2824) mg/day, with 67% (62–73%) exceeding the World Health Organization (WHO) recommended limit of 2000 mg/d. Mean potassium excretion was 1469 (1395–1542) mg/d; < 0.5% met recommended minimum intake levels. Mean sodium-to-potassium ratio was 2.0 (1.9–2.1); not one participant had a ratio that met WHO recommendations. Higher potassium intake and lower sodium-to-potassium ratio were independently associated with age and tertiary education. Sensitivity analyses based on urine creatinine values did not notably alter these findings.

**Conclusions:**

In this first nationally representative study with objective assessment of sodium and potassium excretion in a Caribbean population in over 20 years, levels of sodium intake were high, and potassium intake was low. Younger age and lower educational level were associated with the highest sodium-to-potassium ratios. These findings provide baseline values for planning future policy interventions for non-communicable disease prevention.

## Background

Globally the consumption of sodium exceeds recommended levels [1]; with the main contributor being salt (sodium chloride), which is ubiquitous in the food supply [2]. The World Health Organization (WHO) has set a target of a 30% reduction in mean population salt intake by 2025 (compared with 2010) and the goal of 400 g/day per individual of fruit and vegetables (potassium rich sources), for the prevention of chronic non-communicable diseases (NCDs). In addition, WHO recommends adults consume < 2000 mg sodium and > 3500 mg potassium per day [3, 4]. High sodium diets with inadequate potassium, and high sodium to potassium (Na:K) ratios, are known to be predictors of raised blood pressure (BP) and cardiovascular disease (CVD) mortality [5, 6].

Barbados is an independent, densely populated, Caribbean island (277,821 persons, land area 166 sq. miles), 94% of whose population is of African origin [7]. There is a high prevalence of hypertension (41%) among Barbadian adults (≥ 25 yrs) [8], and 80% of deaths are attributed to NCDs, with 24% of deaths in women and 29% in men being due to CVD, predominantly stroke and coronary artery disease [9]. Data on dietary sodium and potassium intake in the Barbadian population are scarce. The collection of a timed 24 h urine sample for the measurement of sodium and potassium excretion, as proxies for dietary intake, is the method of choice for assessing population levels [10–12], with approximately 85–90% of dietary sodium and > 85% of dietary potassium being excreted in the urine [12, 13].This methodology was last used in Barbados over 20 years ago [14, 15]. However, neither of these two previous studies used representative population-based samples. In fact, as far as we are aware there has not been a population- based study of sodium excretion in the whole of the Caribbean for over 20 years [16].

Our aim in this study was to estimate urinary sodium and potassium excretion in a representative population-based sample of Barbadian adults, and to identify the major dietary sources of sodium. The findings from this study will be used to inform, and provide a baseline for, interventions aimed at improving diet to prevent NCDs in Barbados, and will be of interest throughout the Caribbean.

## Methods

### Study population

The study population was a sub-sample of the Barbados Health of the Nation (HotN) cross-sectional study, which recruited a representative sample of adults aged ≥25 years (*n* = 1234). Details of the sampling, recruitment and data collection methods in the HotN study have been published elsewhere [8]. From this study, a sample of 441 adults aged 25–64 years was selected, stratified by sex and age-group (25–44 and 45–64 years), with the aim of recruiting at least 100 participants in each group. This sample size was based on the consideration that to detect a difference of 1 g in salt excretion per day (equivalent to 17 mmol or 390 mg sodium) between two groups (with alpha of 0.05 and power of 0.8), and assuming a standard deviation in both groups of 50 mmol of sodium (based on WHO guidance), a minimum of 98 participants per group is required [10].

Participants were eligible if they met the following criteria: no reported history of renal, heart or liver disease or stroke; and had not started diuretics in the previous 2 weeks. Pregnant and lactating women were excluded because of their unique nutritional requirements.

### Data collection

After pilot testing field staff were trained in standardized methods and assessed on their competence and adherence to the study protocol, which was checked throughout the period of data collection. Two face-to-face interviews were held in participants’ homes, between June 2012 and November 2013. Participants were seen within 3 months of their HotN interview. Data on age, sex and educational level were collected, by questionnaire. Education was used as a marker of socioeconomic position, grouped into two categories: less than tertiary education and tertiary education. Tertiary education was defined as post-secondary education including college, vocational and university training.

### 24-h urine collection

Sodium and potassium excretion were measured from a single timed 24-h urine collection, which is acceptable for estimating values in population-based studies [12, 17, 18]. Internationally recommended standard procedures developed by the WHO/PAHO Regional Expert Group for Cardiovascular Disease Prevention were followed [10]. At the first interview, equipment, written instructions and a simplified chart detailing the 24-h urine collection method were given and a convenient day for the urine collection identified. To increase compliance with the strict protocol, a diary in which the timing of the urine collection, any missed urine voids, strenuous activity, illness (specifically vomiting or diarrhoea) and any medication taken during the collection period were recorded.

The urine collection commenced by discarding the first urine void at the beginning of the collection period. This point was noted as the start of the 24-h collection. All urine was then collected from that point onwards until the final void on the following day, at the corresponding 24 h finish time. Urine samples were kept in refrigerated conditions or in a cool, dark place on ice until collection on the day of completion. Transportation to the laboratory was undertaken using insulated bags containing ice packs. Urine samples were analysed using the Roche Hitachi Cobas C311 system ion selective electrode (Roche Diagnostics Ltd., Charles Ave, W. Sussex, RH15 9RY, UK) for sodium and potassium at a laboratory certified by the College of American Pathologists. Creatinine concentrations were assessed by the Roche Hitachi Cobas C311 analyser (Roche Diagnostics Ltd., Charles Ave, W. Sussex, RH15 9RY, UK) using the Jaffe’s reaction with alkaline picrate.

Completeness of urine collection was assessed through volume, adherence to protocol and urinary creatinine. In accordance with guidance from the PAHO, para-aminobenzoic acid (PABA) was not used [10]. Urine samples were excluded if the urine volume was < 500 ml or > 5000 ml, timing of collection fell outside the 20–28 h period or the participant reported missing more than one urine void. If participants reported missing more than one void, a second 24-h urine collection was requested.

If the duration of the collection was not exactly 24 h (but within 20–28 h), urinary sodium, potassium, creatinine, and total volume were normalized to a 24 h period. Urinary creatinine excretion is related to lean body mass and to diet but has substantial intra-individual and inter-individual variation [17]. Nonetheless, using creatinine levels to exclude individuals with presumed incomplete urine collection provides a useful, if imperfect, approach to sensitivity analysis. We used two approaches. Firstly, we excluded individuals if their total 24 h creatinine excretion was less than 4 mmol (455 mg) in women and less than 6 mmol (682 mg) in men. This is in line with recent population-based studies assessing sodium excretion in South Africa [19] and the Netherlands [20]. In addition, we calculated expected creatinine excretion (in mg per day) using the formulae of Joossens and Goboers [21] i.e. 24 x body weight (Kg) in men, and 21 x body weight in women. We then restricted our analyses to those individuals whose creatinine was ≥0.7 of predicted. This cut-point is based on a recent systematic review [22] that concluded this provided, on the basis of the current limited evidence, the most sensitive approach to excluding incomplete urine samples.

### Dietary data

Two non-consecutive, interviewer-administered, 24-h dietary recalls were collected from each participant with the aim to capture the participants’ diet on one week day and one weekend day. The systematic probing of all foods and beverages consumed in the previous 24 h, including supplements, were detailed using the United States Department of Agriculture (USDA) multi-pass method [23]. Information on serving sizes and portions consumed in one sitting were estimated using three-dimensional Nasco food models (Nasco Company, 901 Jamesville Ave, Fort Atkinson, Wisconsin 53,538, USA), standard measuring cups and household utensils. The timing, food source, frequency of consumption, cooking method, seasoning use and recipes were documented.

Listed food items were coded and recorded portions converted into grams. These data were then entered into the nutrition software, Nutribase Pro (version 9, Cybersoft Inc., 2016 E. Muirwood Drive, Phoenix, Arizona, USA). Standardized traditional Barbadian recipes, which were analyzed using the weighed recipe approach, [24] were added to the United States Department of Agriculture (USDA) and Canadian food composition database making this software more culturally appropriate. Foods were placed into food categories based on previous work done in Barbados [25].

### Statistics

The data were weighted to account for the clustered sampling design, to adjust for non-response and to reflect the age and sex distribution of the underlying Barbadian population based on the Barbados Census 2010 [7]. A series of linear regression analyses were used to explore the contribution of age, sex and educational level on sodium and potassium excretion. The appropriateness of using linear regression was determined by examining and confirming the normal distribution of the residuals of the linear regression models. We present 95% confidence intervals (95% CI), along with exact *p* values where appropriate. Statistical significance was accepted as *p* < 0.05.

Cut-points for sodium and potassium intake were based on guidance from two agencies: the American Heart Association (AHA) recommends a sodium intake of no more than 1500 mg/d for people of African origin and no more than 2300 mg/d of sodium and a potassium intake of at least 4700 mg/d for all participants [26]; WHO recommends a sodium intake of no more than 2000 mg/d, and a potassium intake of at least 3500 mg/d [27]. We calculated the urinary Na:K ratio. Based on the above targets, a Na:K ratio of approximately 0.49 and 0.57 is recommended by the AHA and WHO respectively. As described in the results, no individual had a ratio that met the AHA or WHO guidance, and therefore we also used a pragmatic cut-point for the ratio of ≤1 i.e. where the potassium excretion (in mg) is equal to or greater than the sodium excretion.

All statistical analyses were performed using Stata software package (V.12, Stata Corp, College Station, Texas, USA).

## Results

A total of 441 participants were selected from the HotN study sample and assessed for eligibility, of these 368 (83%) consented to take part. Four urine samples were deemed incomplete on the basis of total sample volume and excluded. The characteristics of participants in the final study sample of 364 are shown in Table 1. Using WHO body mass index (BMI) cut-points of 25 and 30 kg/m^2^ to classify overweight and obesity, respectively, 66% of participants were classified as overweight or obese and 36% as obese (25% of men, 46% of women were obese). The prevalence of hypertension was 34%, being similar for both sexes. An estimated 9% self-reported a diagnosis of diabetes whilst 13% of the sample were diagnosed with diabetes presenting with a fasting blood glucose level of ≥7.0 mmol/l.

**Table 1:** Characteristics of the Barbados National Salt study population (2012–2013). Unless indicated otherwise, the figures in parentheses are 95% confidence intervals

	Women (*n* = 203)	Men (*n* = 161)	Overall (*N* = 364)
Number (%) by age (years)			
25–44	90 (46.0)	76 (49.1)	16 (47.8)
45–64	113 (54.0)	85 (50.9)	198 (52.2)
Number (%) by education			
< Tertiary^a^	127 (77.9)	99 (76.3)	226 (77.2)
Tertiary^a^	76 (22.1)	62 (23.7)	138 (22.8)
Anthropometry			
Mean BMI (Kg/m^2^)	30.3 (29.0, 31.6)	27.1 (26.0, 28.2)	28.7 (27.9, 29.6)
Mean waist circ (cm)	94.7 (91.9, 97.4)	92.4 (89.7, 95.1)	93.6 (91.9, 95.2)
% overweight (BMI ≥ 25)	73.9 (64.1, 81.8)	57.9 (47.2, 67.9)	66.0 (58.4, 72.9)
% obese (BMI ≥ 30)	46.4 (38.4, 54.6)	25.3 (19.0, 32.8)	36.0 (30.8, 41.5)
Blood pressure			
Systolic BP (mm Hg)	124.4 (121.6, 127.2)	130.5 (127.4, 133.7)	127.3 (125.3, 129.4)
Diastolic BP (mm Hg)	78.1 (76.1, 80.0)	79.5 (77.1, 81.8)	78.7 (77.3, 80.2)
% reported hypertension	25.8 (17.8, 36.0)	14.4 (9.6, 21.0)	20.4 (15.5, 26.3)
% total hypertension	35.5 (27.5, 44.4)	32.7 (23.2, 43.8)	34.1 (27.7, 41.2)
Glucose			
Fasting (mmol/l)	5.5 (5.2, 5.8)	5.4 (5.2, 5.6)	5.4 (5.3, 5.6)
% reported diabetes	9.9 (5.7, 16.5)	9.0 (5.0, 15.6)	9.4 (6.6, 13.3)
% all diabetes	15.1 (9.8, 22.5)	10.9 (6.1, 18.7)	13.1 (9.6, 17.6)

^a^Tertiary refers to all post-secondary school education, including technical and vocational training as well as university course

Percentages are weighted to compensate for unequal probabilities of selection (selecting one individual from household) and for non-response

In Table 2 the mean values (with 95% CI) of sodium and potassium excretion and Na:K ratio, are shown by age and sex. Mean sodium excretion was 2656 mg/d (equivalent to 6.75 g salt/d); and significantly higher in men compared to women (difference 423 mg/d; 95% CI 86–760; *p* = 0.015). Mean potassium excretion was 1469 mg/d, being non-significantly higher in men (1558 mg/d) than women (1386 mg/d); all participants of both sexes and across all age groups were below the minimum recommended potassium intake for adults of 4700 mg/d (the AHA recommended level); with only two participants exceeding 3500 mg/d (the WHO recommended level). The mean Na:K ratio was 2.0. The lowest ratio was 0.79, and thus no participant had a ratio in line with WHO and AHA recommended relative intakes of sodium and potassium. The ratio was greater than 1 for 87% of the population.

**Table 2:** Twenty-four hour urinary sodium and potassium, and the sodium-to-potassium ratio, by age and sex, Barbados (2012–2013)

	Sodium (mg)	Potassium (mg)	Na:K ratio
Women			
25–44 years	2596 (2224, 2968)	1264 (1141, 1386)	2.1 (1.9, 2.3)
45–64 years	2302 (2075, 2530)	1515 (1356, 1673)	1.7 (1.5, 1.8)
Total	2453 (2195, 2710)	1386 (1275, 1497)	1.9 (1.8, 2.0)
Men			
25–44 years	2971 (2681, 3261)	1507 (1372, 1642)	2.1 (2.0, 2.3)
45–64 years	2768 (2489, 3047)	1617 (1421, 1812)	2.0 (1.7, 2.3)
Total	2876 (2665, 3087)	1558 (1442, 1674)	2.1 (1.9, 2.3)
Men and women			
25–44 years	2780 (2544, 3015)	1382 (1290, 1475)	2.1 (2.0, 2.3)
45–64 years	2521 (2322, 2721)	1563 (1440, 1684)	1.8 (1.7, 2.0)
Total	2656 (2488, 2824)	1469 (1395, 1542)	2.0 (1.9, 2.1)

Figures are means (95% confidence intervals)

**Fig. 1:**
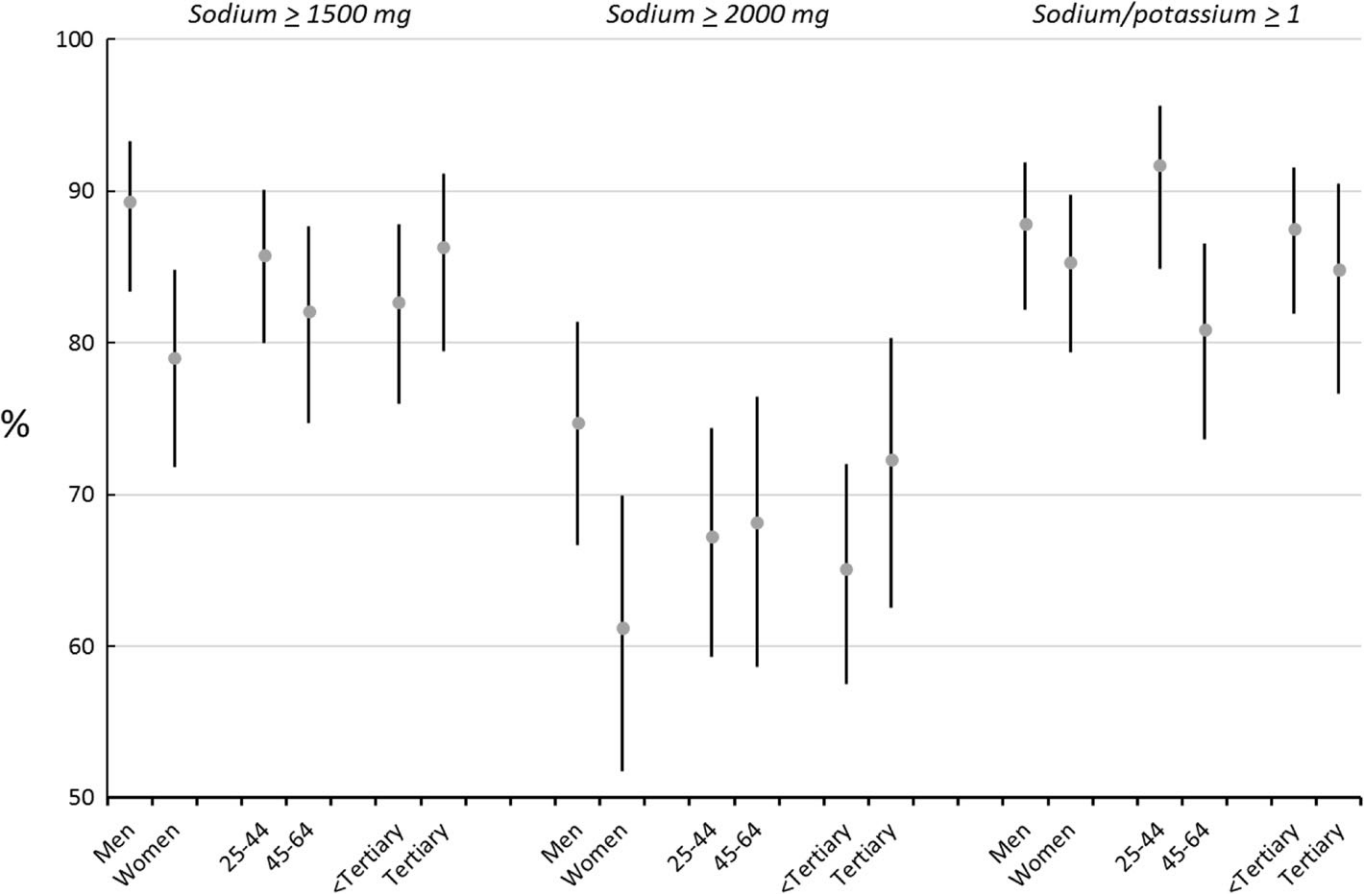
Percentages (95% CIs) exceeding recommended daily levels of sodium excretion and a sodium-to-potassium ratio > 1, by sex, age group and educational level in Barbados (2012–2013). ^a^The AHA recommends a sodium intake of no more than 1500 mg/d for people of African origin. ^b^The WHO upper limit for sodium intake of 2000 mg/d (equivalent to 5 g of salt). ^c^Tertiary refers to all post-secondary school education, including technical and vocational training and university courses

**Fig. 2:**
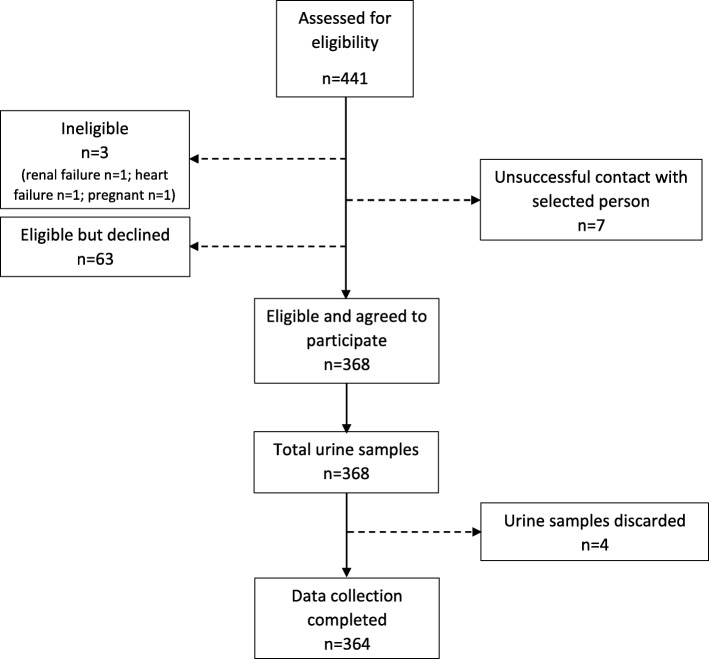
Participant flow chart

The percentages (with 95% CI) of the population consuming more than recommended levels of sodium, using the AHA and WHO guidelines are shown in Figs. 1 and 2**.** The AHA upper limit of 1500 mg was exceeded by 79% women and 89% men. The WHO upper limit of 2000 mg/d (equivalent to 5 g of salt) was exceeded by 68% of the population. Although confidence intervals are broad, there are higher proportions exceeding sodium intake guidelines in those with tertiary education compared with those with less than tertiary education. The proportion of participants exceeding a Na:K ratio of 1 was higher in men, also in the younger age group (25–44 years) and in those participants with a primary or secondary education only.

The results of multiple linear regression are shown in Table 3. Male sex (*p* < 0.014), but neither age nor education, was significantly related to sodium excretion. There was a significant positive association between potassium excretion and age (*p* = 0.002); as well as between potassium excretion and educational level (*p* = 0.024). There was a significant negative association between the Na:K ratio and age (p = 0.002); as well as educational level (*p* = 0.039). Further adjustment for BMI (data not shown) made little difference to these findings.

**Table 3:** Age, sex and educational level^a^ as predictors of sodium and potassium excretion, in multiple linear regression

	Beta (95% CIs)	*P* value
Sodium (mg per day)		
Age (years)	−7.4 (−26.3, 11.4)	0.431
Sex (male vs. female)	428.0 (91.3, 764.6)	0.014
Education (tertiary vs. less than)	−24.7 (− 394.1, 344.7)	0.893
Potassium (mg per day)		
Age (years)	13.3 (5.3, 21.3)	0.002
Sex (male vs. female)	162.3 (−11.8, 336.4)	0.067
Education (tertiary vs. less than)	183.5 (24.9, 342.1)	0.024
Sodium-to-potassium ratio		
Age (years)	−0.032 (− 0.052, − 0.012)	0.002
Sex (male vs. female)	0.331 (− 0.019, 0.682)	0.063
Education (tertiary vs. less than)	−0.427 (− 0.832, − 0.022)	0.039

^a^Analyses by age, sex and educational level entered together

Sensitivity analyses were performed, using urinary creatinine to assess for sample completeness. Firstly, restricting to those with ≥4 mmol (women) and 6 mmol (men) creatinine in their urine (*n* = 317) and then restricting to those with a creatinine index of ≥0.7 (*n* = 184). In both sensitivity analyses, the mean estimates of sodium and potassium excretion were modestly higher, but the 95% CI from these restricted analyses and those of the whole sample overlapped greatly (as shown in Table 2). The Na:K ratio essentially remained unchanged (Tables 5 and 6 in Appendix).

Food groups previously described for Barbados were used to classify foods [25]. The top three food groups for sodium were rice, poultry and bread. (Table 4) Five food items contributed over one-quarter of total sodium dietary intake (28.9%), similar for both sexes. These foods were any bread (9.2%), rice and peas (6.2%), baked chicken (4.9%), macaroni pie (4.3%), white rice (4.3%) (data not shown). No statistically significant differences were found in sodium intake sources for either the top 10 food groups or top 10 food items by age, sex or education.

**Table 4:** Top 10 food group sources of dietary sodium for men and women, by % sodium

Food group	Frequency^a^ (%)	% Sodium (95% CI)
Rice	389 (4.6)	13.4 (13.4, 13.5)
Poultry	487 (5.8)	13.0 (12.9, 13.0)
Bread	472 (5.6)	12.3 (12.2, 12.3)
Fish	339 (4.0)	6.9 (6.9, 6.9)
Processed meats	208 (2.5)	6.3 (6.3, 6.4)
Cakes, sweet breads	278 (3.3)	6.1 (6.0, 6.1)
Pasta	187 (2.2)	5.5 (5.5, 5.5)
Vegetables	589 (7.0)	5.5 (5.5, 5.5)
Ground provisions	427 (5.1)	5.3 (5.3, 5.3)
Red meat	222 (2.6)	5.2 (5.1, 5.2)

^a^Frequency: number of times food group was reported, as a percentage of all food items reported

## Discussion

In this nationally representative, population-based study in Barbados, a high prevalence of CVD risk factors (hypertension: 34%, diabetes: 13%, obesity: 36%) co-exist with high intakes of sodium and inadequate potassium intake. The mean daily sodium excretion exceeded the daily recommendations for both sexes, in all age categories. The AHA recommends an upper limit of 1500 mg/d of sodium for populations of African descent; in our study 79% of women and 89% of men exceeded this upper limit. The WHO upper limit of 2000 mg/d for sodium was exceeded by 68%. Potassium excretion was low, with all participants irrespective of age and sex falling below the adult AHA (4700 mg/d) recommendation and the vast majority (> 99%) below the WHO (3500 mg/d) level. Linear regression analyses indicated that potassium intake was higher in persons aged 45–64 years compared with participants 25–44 years and for those of higher education. The WHO recommends a Na:K ratio of < 0.57; if the AHA guidelines are met the ratio would be < 0.32 (1500 divided by 4700). The ratio of Na:K is a critical factor in hypertension. Higher Na:K ratios have been associated with diets high in processed foods, and thus high in sodium, and low in potassium [28, 29].

Within the Caribbean region there have been no recent population-based measurements of sodium excretion using the ‘gold standard’ method of 24-h urinary collection. Only three studies have been conducted within the last 25 years, none of which used a nationally representative sample [16]. The 2010 global estimated mean sodium excretion in adults was 3950 (3890–4010) mg/day; being higher in men than that in women: 4140 (4040–4230) vs 3770 (3690–3850) mg/day, respectively, higher than the mean estimates found in our study [16]. However, our findings are comparable to those described in other parts of the Caribbean, albeit over 20 years ago. For example, the estimated mean sodium excretion levels were1920 mg/d and 2930 mg/d for Jamaica and Trinidad respectively [16].

A limited number of studies have used the 24-h urine collection method to evaluate both sodium and potassium excretion concurrently. Recent work in South Africa, estimated the median sodium and potassium excretion at 2827 mg/d and 1307 mg/d, respectively. The majority (92.8%) of the population did not meet the WHO recommended daily potassium intake of 3500 mg/d, and 65.6% consumed more than 2000 mg/d sodium. The median Na:K ratio was 3.5. Overall, findings of this South African study are similar to ours [30]. In China the mean 24-h urinary sodium excretion level was substantially higher (5336 mg/d) whereas potassium levels were comparable (1591 mg) to our estimates in Barbados. Males were also found to have higher urinary sodium excretion than females. The mean Na:K ratio was 6.7 [31]. Our work is to the best of our knowledge, the first in the Caribbean to assess sodium and potassium excretion concurrently.

Our study had several strengths. We used a representative sample, with 83% of those invited participating. Our use of 24-h urine collection, the clinical “gold standard” method, provided an objective measurement of electrolyte excretion as a proxy for dietary intake. We followed strict protocols and comprehensive instruction to ensure completeness of urine collection as laid out in the WHO guidelines [10], similar to other large population-based studies [32, 33]. In addition to the application of the strict urine collection protocol, the sensitivity analyses using urinary creatinine measurements provided reassurance that population mean values are not significantly biased by incomplete urine collection. By recording two non-consecutive dietary recalls, we obtained a more representative picture of an individual’s usual diet.

A limitation of our study is the use of a single 24-h urine collection per individual. This is a valid and reliable approach to estimating population levels of sodium and potassium intake [10–13] but is poor at an individual level because of marked day to day variation [12]. In addition, our sample size was relatively small. With the limited resources available, we had the power to detect approximately a 1 g salt difference between two subgroups, such as males versus females, younger (25–44 years) and older (45–65 years) age groups and higher versus lower education. However, given the single 24-h urine collection per individual and the relatively small sample size our study was not designed to evaluate the relationships between sodium and potassium intake and blood pressure.

The study methods rely on the abilities of individuals to completely and accurately collect their urine over 24 h. The robustness of our findings was tested through sensitivity analyses using urinary creatinine levels to estimate whether complete urine collection was achieved. This is an imperfect approach for several reasons, including marked day to day intra and inter individual variability in creatinine excretion [17, 34], and that the equations used to estimate expected excretion have not been validated in our population. We used two complementary approaches, one that excluded 13% of our sample and the other excluding almost half. The latter was based on a creatinine cut off based on a recent systematic review [22] as being the most sensitive to exclude incomplete urine collection, but is likely nonetheless to have excluded considerably more individuals with complete than with incomplete urine collection [22]. We interpret the findings of these sensitivity analyses as suggesting that while bias in the mean estimates from the main sample is possible, correcting for this would not substantially affect our conclusions.

Measurement error in the assessment of dietary intake using 24-h recall may have occurred. However, the use of trained data collectors using food models and household measures to quantify portion size, would have reduced this potential limitation. We appreciate that the underlying USDA and Canadian food composition databases of Nutribase, may have not precisely captured the actual sodium content in the reported Barbadian foods, despite the addition of traditional Barbadian recipes to the database [24]. Most of the dietary recalls were recorded on week days (74%) with all weekdays being evenly represented (11–14%) except for Monday (22%). This may have influenced our findings.

Globally, most countries have adopted a multifaceted approach towards strategies for salt reduction. Food reformulation, consumer education, front of pack labelling (FoPL), interventions in public institution settings (such as schools, hospitals and the workplace) and taxation have been pursued [35]. The most frequently used FoPL schemes are logos and symbols, which also include traffic light style labels, to indicate whether the product meets established nutrient criteria [36]. The 36% reduction in population salt intake achieved in Finland since 1993 is partially attributed to its mandatory warning labels on high salt foods. This in turn led to a significant reformulation of high sodium foods [37, 38]. To date however only two developing countries, South Africa and Argentina, have adopted comprehensive legislative schemes to limit the salt content in foods [39, 40].The main contributors to sodium intake in our study, any bread (9.2%), rice and peas (6.2%), baked chicken (4.9%), macaroni pie (4.3%) and white rice (4.3%), were similar for both sexes. These foods contain sodium mainly added during the cooking process making them sodium dense foods (Table 7 in Appendix). Through public health promotion and education changes in food preparation practices can be achieved [41].

For Barbados to reach recommended WHO and AHA daily targets, potassium excretion would need to double and sodium excretion to decrease by one-third. We have identified the main dietary sources of sodium which could be used as potential targets, in a future population-wide salt reduction campaign. Future qualitative research is warranted to explore the drivers and barriers towards food preferences. A combined effort involving both individual and societal interventions will be needed to address these dietary imbalances and to reduce the high levels of obesity and physical inactivity [42] for this population at high risk of CVD.

## Conclusions

This study, conducted in Barbados, is the first in the Caribbean to provide an objective assessment of sodium and potassium excretion. In this population at high risk of hypertension, sodium excretion was high and potassium excretion low, across all age, sex and educational strata. These findings provide baseline metrics which can be used for designing and evaluating dietary interventions targeted to reduce the risk of hypertension in this and similar populations.

## Appendix

**Table 5:** 24-h urinary sodium and potassium excretion, and sodium-to-potassium ratio, by age and sex: restricted to those with greater or equal to 4 mmol/d (women) and 6 mmol/d (men) creatinine in their urine (n = 317). Figures are means with 95% confidence intervals (95% CI)

	Sodium (mg)	Potassium (mg)	Sodium/potassium
Women			
25–44 years	2623.1 (2227.8, 3018.3)	1279.5 (1143.7, 1415.3)	2.1 (1.9, 2.3)
45–64 years	2472.2 (2222.4, 2722.1)	1594.0 (1422.2, 1765.9)	1.7 (1.5, 1.9)
Total	2555.1 (2275.4, 2834.8)	1421.2 (1296.9, 1545.4)	1.9 (1.8, 2.1)
Men			
25–44 years	3044.8 (2747.3, 3342.3)	1548.2 (1401.8, 1694.7)	2.1 (1.9, 2.3)
45–64 years	2785.1 (2511.6, 3058.7)	1639.4 (1450.8, 1827.9)	2.0 (1.6, 2.3)
Total	2924.8 (2718.0, 3131.7)	1590.3 (1474.1, 1706.5)	2.1 (1.9, 2.2)
Men and women			
25–44 years	2827.2 (2586.2, 3068.2)	1409.5 (1309.0, 1510.1)	2.1 (2.0, 2.3)
45–64 years	2627.3 (2447.0, 2807.6)	1616.5 (1489.7, 1743.2)	1.8 (1.6, 2.0)
Total	2736.0 (2572.2, 2899.8)	1503.9 (1427.5, 1580.4)	2.0 (1.9, 2.1)

**Table 6:** 24-h urinary sodium and potassium excretion, and sodium-to-potassium ratio, by age and sex, restricted to those with ≥0.7 of predicted sex specific creatinine excretion per kg body weight (*n* = 184)

	Sodium (mg)	Potassium (mg)	Sodium/potassium
Women			
25–44 years	3137 (2433, 3841)	1416 (1180, 1652)	2.3 (1.9, 2.7)
45–64 years	2316 (1993, 2638)	1631 (1344, 1918)	1.6 (1.3, 1.9)
Total	2735 (2301, 3169)	1521 (1317, 1725)	2.0 (1.7, 2.2)
Men			
25–44 years	3130.1 (2797, 3464)	1569.7 (1430, 1709)	2.2 (1.9, 2.4)
45–64 years	2800.1 (2546, 3054)	1651.0 (1438, 1864)	1.9 (1.6, 2.3)
Total	2987.6 (2760, 3215)	1604.8 (1489, 1721)	2.1 (1.9, 2.2)
Men and women			
25–44 years	3133 (2797, 3468)	1512 (1400, 1624)	2.2 (2.0, 2.4)
45–64 years	2592 (2388, 2795)	1642 (1471, 1813)	1.8 (1.5, 2.0)
Total	2887 (2673, 3100)	1571 (1473, 1670)	2.0 (1.8, 2.2)

Figures are means with 95% confidence intervals (95% CI)

**Table 7:** Percentage contribution of the top three individual foods within each of the top 10 food groups in Barbados (2012–2013)

Food group	Food item	Proportion %	Sodium density (mg/100 g)
Poultry	Baked chicken	37.8	251
	Fried chicken	26.4	510
	Chicken roti	14.1	287
Rice	Rice and peas	46.4	190
	White rice	31.8	382
	Vegetable rice	9.7	251
Bread	White/whole wheat^a^	30.4	511
	Bakes	13.0	723
	Bran	13.1	472
Fish	Fried fish	45.3	469
	Fish, in oil	21.8	396
	Stewed/steamed	14.8	717
Processed meats	Hotdog	30.5	684
	Ham	19.5	1038
	Luncheon meat	17.1	820
Cakes, sweet breads	Coconut bread	54.6	752
	Cake-Bajan Pudding	15.15.3	403
	Chocolate cake		334
Pasta	Macaroni Pie	79.0	293
	Chow Mein	6.2	439
	Macaroni and cheese	4.2	336
Vegetables	Mixed with salt	31.3	271
	Pickled cucumber	17.77.6	1208
	Coleslaw		270
Ground provisions	French fries/wedges	37.6	757
	English potato	31.9	333
	Mashed/boiled Coucou	9.7	196
Red meat	Beef stew	22.4	480
	Lamb stew	17.6	496
	Ground beef	12.5	576

^a^Whole wheat and white bread contributed the same proportion of sodium within the bread category

## Abbreviations

AHA: American Heart Association
BMI: Body mass index
BP: Blood pressure
CI: Confidence interval
CVD: Cardiovascular disease
FoPL: Front of packet labelling
HotN: Health of the Nation study
K: Potassium
Na:K: Sodium-to-potassium ratio
Na: Sodium
NCDs: Non-communicable diseases
PAHO: Pan American Health Organization
USDA: United States Department of Agriculture
WHO: World Health Organization

## Glossary: Description of some commonly consumed traditional composite dishes and foods in Barbados

Bakes: A flour-based dumpling, which is shallow-fried in oil. Usually eaten as a breakfast item or snack, with fish cakes or on their own.
Chicken and potato roti: Chicken and potato curry wrapped in a roti skin and eaten as a meal.
Coconut bread: Dense cake made with grated coconut, vanilla essence and dried fruit (raisins, cherries). This is a traditional Barbadian delicacy
Coucou: Part of the National dish made from ground corn (cornmeal) and boiled okras, cooked into a firm paste. Usually served with steamed fish and frizzled salt fish
Ground provisions: Name given in the Caribbean to tubular root vegetables which are encased in the ground during growth; such as sweet potatoes, cassava (yucca), eddoes, tania and yams. Plantains, green bananas, corn and breadfruit, although not grown in the ground, are also considered to be ground provisions because they are often cooked with tubular root vegetables. Ground provisions are served as the main carbohydrate of the meal or as a side dish
Macaroni pie: Baked dish of macaroni mixed with a seasoned cheese-based sauce, a popular Barbadian starchy food dish
Minced meat: Sautéed ground beef, seasoned with onions, tomatoes and herbs. May be served with macaroni, chow mien or rice as part of a main meal
Peas and rice: A mixed dish of dried or fresh legumes (kidney beans, lentils, black-eye peas) and parboiled rice. Boiled in salted water seasoned with onion and herbs until dry. Salted cured meat may also be added during boiling. This dish is a staple in the Barbadian diet
Vegetable rice: A mixture of seasonal vegetables, most commonly carrots, beans or spinach, cooked with parboiled rice to form this composite dish. It is eaten as a starchy food.
